# Perceptions of Residents among Rural Communities with Medical Group Practice in Japan

**DOI:** 10.3390/ijerph16245124

**Published:** 2019-12-15

**Authors:** Toshie Manabe, Tsutomu Sawada, Takao Kojo, Seitaro Iguchi, Sanae Haruyama, Takahiro Maeda, Kazuhiko Kotani

**Affiliations:** 1 Division of Community and Family Medicine, Center of Community Medicine, Jichi Medical University, Shimotsuke-City, Tochigi 329-0498, Japan; manabe@jichi.ac.jp; 2 Kochi Health Sciences Center, Kochi 781-8555, Japan; t-sawada@mvc.biglobe.ne.jp; 3 Department of Health Management, School of Health Studies, Tokai University, Kanagawa 252-0331, Japan; kozzy@tsc.u-tokai.ac.jp; 4 Department of Community Medicine, Niigata University Graduate School of Medicine and Dental Sciences, Niigata 951-8122, Japan; seita.iguchi@me.com; 5 Faculty of Nursing, Jichi Medical University, Tochigi 329-0498, Japan; sharu@ms2.jichi.ac.jp; 6 Department of General Medicine, Nagasaki University Graduate School of Biomedical Science, Nagasaki 852-8523, Japan; tmaeda@nagasaki-u.ac.jp

**Keywords:** rural health, physician shortage, nurse practitioner, remote consultation, family structure

## Abstract

Elucidating the perceptions of residents regarding medical group practice (GP) among rural communities (GP-R) in Japan will be useful for establishing this system in such communities. A survey by questionnaire, as made by experts in rural health, was conducted in 2017. The self-administered questionnaire inquired about the perceptions of residents for accepting the GP-R into the community’s healthcare using seven major elements of GP-R. The questionnaire was randomly distributed to 400 adult residents who lived in rural communities with a recently launched GP and had access to clinics within the communities. Among the 321 respondents, comparisons were made between younger (≤sixties) and older (≥seventies) residents, and a stepwise multiple regression analysis was performed to extract the factors influencing acceptance of the GP-R system. The results showed that older residents had a greater disapprove of being treated by different physicians daily or weekly in clinics (*p* < 0.001) and the use of telemedicine (*p* < 0.001) compared with younger residents. Younger residents showed a greater disapproval of clinics closing on weekdays than older residents (*p* = 0.007). Among all respondents, regardless of age groups, over half of residents approved of the involvement of nurse practitioners in the GP-R. Living with family and children was also extracted as an independent factor influencing a positive perception of the GP-R. These data suggest that the promotion of GP-R should consider generation gaps in the approach to medical practice as well as the family structures of residents. The involvement of nurse practitioners can also encourage the acceptance of GP-R in Japan.

## 1. Introduction

At At the forefront of global aging, Japan has been experiencing a more rapidly aging population than any other country [1]. The Ministry of Health, Labour and Welfare of Japan estimates that 30% of the total population will be over 65 years old by 2030 [2]. This population aging is thought to be partly due to the public health insurance system covering the entire populace of Japan [1,2,3], which influences the health and long-term care of the citizens. Therefore, the government proposed establishing a community-based integrated care system with the purpose of comprehensively ensuring the provision of medical/health care, nursing care, preventive care, housing, and livelihood support [3].

However, the aging of the population has caused problems in rural and/or remote mountains, islands, and peninsulas, where approximately 10% of the Japanese population reside [4]. Such communities have long had various issues associated the medical/health care systems, including relocation of physicians’ practices, aging physicians, a lack of 24-h availability of medical care, little nursing and home care [5], few public services [6], and little social and family support [7]. In addition, physicians working in rural communities have been reported to be dissatisfied with their work and lifestyle [8].

Medical group practice (GP) in rural communities (GP-R), a system in which clinics are shared by several physicians, is recognized as a strategic option for overcoming these issues [9]. Unlike in the GP system that is affiliated with a large hospital, physicians who work at various clinics (or hospitals) in the GP-R system can provide medical care for many patients in wide communities with an expanded range of medical fields [9]. The GP-R system will lead to the use of telemedicine and involvement of nurse practitioners in providing medical care to patients on behalf of physicians. People who have previously received traditional medical care at a face-to-face, patient-centred, physician-led service level may show resistance to the new GP-R system. As the availability of a sufficient number of physicians may be difficult to achieve even with the GP-R system, at times when there are no physicians available in rural communities on weekdays, holidays, or at night. With the introduction of GP-R into some rural communities of Japan, its level of acceptance by residents of these communities seemingly needs to be clarified.

Understanding the feelings of residents toward a new system will be useful for establishing such a system in a community. The aim of the present study was to evaluate the perceptions of the GP-R system among residents in rural communities of Japan where the system was recently launched. As the perceptions of medical/health care systems can differ among age/generation groups [10], the analysis was also considered with age-based stratification.

## 2. Methods

A cross-sectional survey was conducted in 2017 using a self-administered questionnaire to assess the perceptions of GP-R in regions of Japan where the system had recently been launched (Gifu (seven clinics as a group), Niigata (three clinics as a group), Shizuoka (two clinics as a group), and Kochi (five clinics as a group) Prefectures). The inclusion criteria of respondents were residents living in the surveyed communities, ≥20 years old, with access to the clinics within the communities. Residents were users of the clinics for any health problems. The questionnaire was randomly distributed to residents by clinic staff. The questionnaire was originally designed by the study investigators (experts in rural health) to address the primary elements (seven questions) constituting the GP-R [11] (Table 1). All questions were either closed-ended or answered using a five-point Likert-type scale. For the scaled questions, five and four points were assigned for responses of “strongly approve” or “approve”, respectively, indicating a positive perception of GP-R; 3 points were assigned for responses of “undecided”, and two points and one point were assigned for “disapprove” or “strongly disapprove”, respectively, indicating a poor perception of GP-R.

Data were reported as the frequency with percentages for categorical variables and as the mean with standard deviations for continuous variables. The sample size was set in order to detect an effect of 0.2 with a power of 0.8, even after 15% of dropout. Comparisons were made between younger (≤sixties) and older (≥seventies) residents using the χ^2^ test, Fisher’s exact test, or Student’s *t*-test. Scores for positive perceptions of the GP-R were calculated as an attitude score, in accordance with responses, using a factor analysis adjusted to give a total score of 10. Scores were compared between the two age groups. To determine the possible factors influencing a higher attitude score (positive perception of approval) in all respondents, a stepwise selection method was used to select variables for a multiple regression analysis using residents’ backgrounds as the baseline adjustment covariance, including the age, gender, living condition, and condition of underlying diseases. The data analyses were performed using the IBM SPSS software program, version 25.0 (IBM Corp., Armonk, NY, USA). In all analyses, significance levels were 2-tailed, and *p* values < 0.05 were considered to indicate a statistical significance.

This study was approved by the institutional ethics committee (17-142). Written informed consent was obtained from all participants.

## 3. Results

### 3.1. General Characteristics

Of the 400 residents included in the present survey, a total of 321 (response rate: 80%) were respondents. Of these, 39.1% were male, and 53.9% were ≥70 years old. Table 2 shows the general characteristics of the study participants. Most residents in the younger group lived with their family, including children, whereas most older residents lived with their spouse (*p* < 0.001). The mean number of underlying diseases in the older group was 2.0, which was significantly different from that in the younger group (*p* < 0.001). The prevalence of residents with no underlying diseases was 64.7% and 49.3% in the older and younger groups, respectively (*p* = 0.007).

### 3.2. Perceptions of the GP-R

The perceptions of GP-R by generation are described in Table 3. The prevalence of residents who approved of medical care being provided by different physicians each day or week was similar in the younger (49.7%) and older (48.8%) groups. The prevalence of residents who disapproved of this practice was 34.5% in the older group and 15.0% in the younger group (*p* < 0.001). More residents in the younger group disapproved than approved of clinics closing on a weekday, while more residents in the older group approved than disapproved of this practice (*p* = 0.007). Although 50.7% of younger residents were undecided about using telemedicine versus a face-to-face consultation with a physician, 48.5% of older residents disapproved of telemedicine (*p* < 0.001). Nurse practitioners were accepted at a high prevalence by both younger (57.2%) and older (53.3%) residents, with no significant difference between the groups.

As described in Table 3, the mean attitude scores were not significantly different between the younger and older groups (*p* = 0.127). In a multivariate analysis of all residents, living with family and children was an independent factor influencing attitude scores that indicated a positive perception of the GP-R system, as shown in Table 4.

## 4. Discussion

In the present study, differences in perceptions of the GP-R system were revealed among different generations of residents in our surveyed communities. In this context, there were significant differences in perceptions about GP-R for three elements: the provision of medical care by different physicians each day or week, clinics closing on a weekday, and the use of telemedicine. There was a high approval of nurse practitioners among residents, regardless of generation. Living with family and children was also found to be a factor influencing a positive perception of the GP-R system.

Regarding the rotation of physicians on a daily or weekly basis in GP-R, approximately half of residents in each group approved of this practice; however, older residents disapproved of this more than younger ones. This may be related to the general assumption that older residents are used to or prefer a more traditional, face-to-face patient–physician relationship [12].

The finding of greater disapproval of clinics closing on a weekday among younger residents might be partly accounted for by a fear of acute illness among respondents’ children. Unlike older residents, who mainly access the clinics for their own problems with chronic diseases, younger residents with children typically access clinics more often for acute illnesses, as a healthcare-seeking behavior. A similar finding was reported in a Bangladesh study, indicating that the daily availability of physicians, type and severity of disease, and child’s age can be important factors affecting parents’ healthcare-seeking behavior in rural communities [13]. Furthermore, approximately half of residents in both the younger and older groups answered that an emergency treatment at night would be problematic in the GP-R system [13]. The 24-h availability of medical care for not only younger but also older people [14,15] is a general issue with the GP-R system.

The lower approval of telemedicine in older residents was speculated to be due to their perception of this type of healthcare being technically difficult to use or their preference for receiving a direct clinical consultation with a physician. These explanations may be supported by a previous observation that people with chronic diseases are more likely to prefer face-to-face contact with a physician [12]. Indeed, in the present study, considerably more residents in the older group had hypertension and a history of cerebral infarction than in the younger group.

Over half of both younger and older residents approved of receiving medical care from a nurse practitioner instead of a physician. The role of nurse practitioners in Canada was originally intended to meet the medical/healthcare needs of underserved populations residing in remote rural areas in northern parts of the country [16]. According to this concept [16], nurse practitioners in rural communities of Japan can assist with medical care on behalf of physicians, thereby helping physicians enhance their work and lifestyle [17,18]. At present, nurse practitioners have been serving in many roles in a variety of medical/healthcare settings, such as prescribing practices [19], chemotherapy [20], and memory assessment services for people with suspected dementia [21]. A recent study in England suggested that nurse practitioners can help achieve equivalent clinical outcomes to general practitioners when performing out-of-hours urgent primary care [22]. Establishing trust in the patient–nurse relationship is essential for the spread and acceptance of nurse practitioners [23]. Nurse practitioners have not yet been introduced to the GP-R system in Japan, in contrast to the relative activity of nurse practitioners in overseas settings. We believe that nurse practitioners and similar professionals will help overcome critical issues of providing medical care in rural communities in terms of the rotation or relocation of physicians’ practices as well as difficulty in ensuring the 24-h availability of medical care once residents become informed of the quality of nurse practitioners’ care.

Living with family and children was extracted as a factor for the positive acceptance of GP-R, indicating the importance of considering family structure when promoting GP-R. The general care of older people has traditionally been a family responsibility in Japan. However, changes in the family size and function have led to having fewer children and fewer family members being available to provide care for older people [24]. A previous study in Japan reported that older people who lived with a daughter had a reduced risk of being admitted to a geriatric institution [25]. Another study in rural communities of Japan showed that men who live alone have a high risk of frailty [26]. Living with other family members means better safety and security for the health of older residents, as well as for all family members, possibly leading to a better acceptance of a new medical system like GP-R.

Several limitations associated with the present study warrant mention. First, most measures relied on self-reporting by residents. Second, all residents did not necessarily respond to the survey. For the scaled questions, not simply yes/no options but neutral options (which might be chosen without contemplation in some cases) were allowed. The possibility of bias in responses therefore cannot be overlooked. Third, we did not perform any comparison of our findings with those of residents in rural communities without a GP-R system. Fourth, the residents’ social data (e.g., occupation, working sites and hours) were not examined, although these are associated with visits to clinics. Despite these limitations, given the current dearth of information on perceptions of residents regarding GP-R, the present study will help expand the GP-R system to other rural communities of Japan. Furthermore, this will provide internationally valuable information from Japan, the most rapidly aging country in the world [1], for considering rural healthcare in countries worldwide, despite a difference in medical care systems across borders [3].

## 5. Conclusions

The present study on the perceptions of residents of Japan concerning GP-R offered several new insights. These data suggest that gaps in perceptions between generations as well as the characteristics of certain family structures should be taken into account when incorporating GP-R into the healthcare system of rural communities in Japan. The incorporation of nurse practitioners is also an important item to consider. Further research is warranted to confirm the acceptance of the GP-R system.

## Figures and Tables

**Table 1:** Questions for the perception of residents regarding the seven major elements on medical group practice in rural communities.

No.	Questions
Q1	What do you think about the medical/healthcare system in which different physicians are in charge on a daily or weekly basis?
Q2	What do you think about the clinics being closed on weekdays?
Q3	What do you think about the emergency treatment at night or on holidays?
Q4	What do you think about needing medical care for specialized departments, such as ophthalmology, otolaryngology, obstetrics, gynecology, dermatology, or orthopedics?
Q5	Is the current healthcare system sufficient to allow you to live in your home until the end of your life?
Q6	What do you think about receiving remote consultations via telemedicine instead of face-to-face consultations with physicians?
Q7	What do you think about qualified nurse practitioners treating you on behalf of physicians, such as administering drugs and treating injuries?

**Table 2:** General respondents’ characteristics.

	Younger Group (≤60s: *n* = 148)	Older Group (≥70s: *n* = 173)	*p* Value
Gender, n (%)			0.909
Male	57 (38.5)	68 (39.3)	
Female	91 (61.5)	105 (60.7)	
Age/generation, n (%)			-
20s	4 (2.7)	-	
30s	8 (5.4)	-	
40s	18 (12.2)	-	
50s	29 (19.6)	-	
60s	89 (60.1)	-	
70s	-	89 (51.4)	
≥80s years old	-	84 (48.6)	
Living condition, n (%)			<0.001
Live alone	10 (6.8)	26 (15.2)	
Live with spouse	41 (28.1)	93 (54.4)	
Live with family (no children)	32 (21.9)	11 (6.4)	
Live with family (with children)	63 (43.2)	41 (24.0)	
Underlying diseases, n (%)			
Number of underlying disease—mean (SD)	1.2 (1.5)	2.0 (2.1)	<0.001
Cerebral infarction	1 (0.7)	10 (5.8)	0.013
Hypertension	35 (23.5)	66 (38.2)	0.006
Dyslipidemia	6 (4.0)	11 (6.4)	0.456
Diabetes mellitus	12 (8.1)	18 (10.4)	0.565
Other orthopedic diseases	6 (4.0)	13 (7.5)	0.238
Malignant	3 (2.0)	4 (2.3)	1.000
others	8 (5.4)	19 (11.0)	0.105
No underlying diseases	73 (49.3)	112 (64.7)	0.007
Long-term care, n (%)			
Receive LCT	0 (0.0)	12 (7.1)	0.001
Provide LCT for family member	34 (23.1)	21 (12.9)	0.025

SD, standard deviation; LCT, long-term care insurance program.

**Table 3:** Perceptions of medical group practice in rural communities.

	Younger Group (≤60s: *n* = 148)	Older Group (≥70s: *n* = 173)	*p* Value
Medical care provided by different physicians every day or week (*n* = 315)			<0.001
Disapprove	22 (15.0)	58 (34.5)	
Approve	73 (49.7)	82 (48.8)	
Neither disapprove nor approve	52 (35.4)	28 (16.7)	
Closing the clinic on a weekday (*n* = 317)			0.007
Disapprove	65 (44.2)	61 (35.9)	
Approve	40 (27.2)	75 (44.1)	
Neither disapprove nor approve	42 (28.6)	34 (20.0)	
Emergency treatment at night or on a holiday (*n* = 311)			0.611
Disapprove	70 (48.6)	80 (50.0)	
Approve	36 (25.0)	45 (28.1)	
Neither disapprove nor approve	38 (26.4)	35 (21.9)	
Needing specialized medical care (*n* = 311)			0.426
Disapprove	55 (37.7)	74 (44.8)	
Approve	56 (38.4)	54 (32.7)	
Neither disapprove nor approve	35 (24.0)	37 (22.4)	
Evaluating the current medical care system for the death at your home (*n* = 302)			0.491
Disapprove	34 (24.3)	34 (21.0)	
Approve	46 (32.9)	64 (39.5)	
Neither disapprove nor approve	60 (42.9)	64 (39.5)	
Having telemedicine instead of face-to-face medical consultation (*n* = 309)			<0.001
Disapprove	38 (26.4)	80 (48.5)	
Approve	33 (22.9)	19 (11.5)	
Neither disapprove nor approve	73 (50.7)	66 (40.0)	
Getting medical care or receiving drugs from a nurse practitioner instead of a physician in the future			0.159
Disapprove	12 (8.3)	26 (15.4)	
Approve	83 (57.2)	90 (53.3)	
Neither disapprove nor approve	50 (34.5)	53 (31.4)	
Attitude scores, mean (SD)	6.03 (1.19)	5.80 (1.50)	0.127

SD, standard deviation.

**Table 4:** Extracted factor influencing a positive perception of medical group practice in rural communities.

	Coefficient	SE	*p* Value	95% CI
Live with family and children	0.472	0.224	0.036	0.031–0.912

SE, standard error; CI, confidence interval.
